# A188 FACTORS ASSOCIATED WITH CLINICAL REMISSION IN PEDIATRIC LUMINAL CROHN’S DISEASE: A RETROSPECTIVE COHORT STUDY

**DOI:** 10.1093/jcag/gwab049.187

**Published:** 2022-02-21

**Authors:** S Sassine, S Fadela Zekhnine, M Qaddouri, L Djani, C Cambron-Asselin, M Savoie-Robichaud, Y Lin, K Grzywacz, V Groleau, M Dirks, É Drouin, U Halac, V Marchand, C Girard, O Courbette, N Patey, D Dal Soglio, C Deslandres, P Jantchou

**Affiliations:** Centre Hospitalier Universitaire Sainte-Justine Centre de Recherche, Montreal, QC, Canada

## Abstract

**Background:**

The natural evolution of Crohn’s disease is incompletely understood in the pediatric population. Data on factors influencing time-to-remission are very limited in the literature.

**Aims:**

The aim of this retrospective cohort study was to describe the time to clinical remission in children with Crohn’s disease as well as changes over the past decade and to identify factors associated with time to clinical remission.

**Methods:**

Patients under 18 years old diagnosed between 2009 and 2019 were included. All data were collected from the patients’ medical records and the CHU Sainte-Justine inflammatory bowel disease registry. Survival analyses and linear regression models were used to assess the impact of clinical, laboratory, endoscopic, histological and therapeutic factors on time to clinical remission.

**Results:**

654 patients were included in the study. There was no change in the time to clinical remission over the past decade. Female sex in adolescents (ajusted bêta regression coefficient (aβ)= 31.8 days, p= 0.02), upper digestive tract involvement (aβ= 46.4 days, p= 0.04), perianal disease (aβ= 32.2 days, p= 0.04), presence of active inflammation on biopsies (aβ= 46.7 days, p= 0.01) and oral 5-ASA exposure (aβ=56.6 days, p= 0.002) were all associated with longer time to clinical remission. However, antibiotic exposure (aβ= -29.3 days, p=0.04), increased eosinophils on biopsies (aβ= -29.6 days, p=0.008) and combination of exclusive enteral nutrition and TNF- alpha inhibitors as induction therapy (aβ= -36.8, p=0.04) were associated with shorter time to clinical remission.

**Conclusions:**

Time to clinical remission did not improve during the decade and was associated with baseline clinical and histological data and treatment strategies. Combination of enteral nutrition and TNF-alpha inhibitors was associated with faster clinical remission.

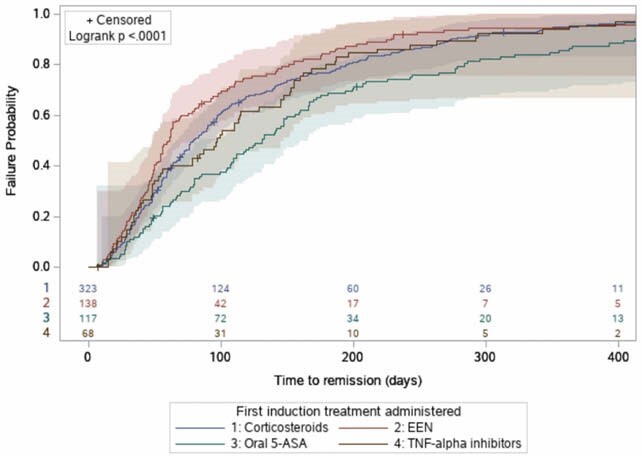

Kaplan-Meier curve representing the time to clinical remission of patients according to the first induction treatment administered.

**Funding Agencies:**

NoneFonds Recherche Santé Québec / Fondation du CHU Sainte-Justine

